# Serum level of high sensitive C-reactive protein and IL − 6 markers in patients with treatment-resistant schizophrenia in Ethiopia: a comparative study

**DOI:** 10.1186/s12888-021-03443-4

**Published:** 2021-08-31

**Authors:** Feyissa Challa, Daniel Seifu, Meron Sileshi, Tigist Getahun, Zeleke Geto, Desta Kassa, Melkam Alemayehu, Miraf Mesfin, Abebaw Fekadu, Yimtubezinash Woldeamanuel

**Affiliations:** 1 National References Laboratory for Clinical Chemistry, Ethiopian Public Health Institute, Addis Ababa, Ethiopia; 2 Division of Basic Sciences, University of Global Health Equity, Kigali, Rwanda; 3 HIV/AIDS and Tuberculosis Research Directorate, Ethiopian Public Health Institute, Addis Ababa, Ethiopia; 4 Department of Psychiatry, School of Medicine, College of Health Sciences, Addis Ababa University, Addis Ababa, Ethiopia; 5 Centre for Innovative Drug Development and Therapeutic Trials for Africa (CDT-Africa), College of Health Sciences, Addis Ababa University, Addis Ababa, Ethiopia; 6 Global Health & Infection Department, Brighton and Sussex Medical School, Brighton, United Kingdom; 7 King’s College London, Centre for Affective Disorders, Department of Psychological Medicine, Institute of Psychiatry, Psychology and Neuroscience, London, United Kingdom; 8 Department of Microbiology, Immunology, and Parasitology, School of Medicine, College of Health Sciences, Addis Ababa University, Addis Ababa, Ethiopia

**Keywords:** Schizophrenia, Interleukin-6 (IL-6), High sensitive C - reactive protein (hsCRP), Inflammatory markers, Ethiopia

## Abstract

**Background:**

Accumulating evidence indicates that schizophrenia is accompanied by significant activation of the immune system; however, there is limited data from low and middle-income countries (LMIC). Inflammatory markers may be more relevant in LMIC settings where infectious conditions are more prevalent and may thus play some role in the causation and maintenance of schizophrenia. The aim of this study was to assess the level of inflammatory markers high sensitive C-reactive protein (hsCRP) and interleukin-6 (IL-6) in patients with schizophrenia.

**Materials and methods:**

The study population consisted of a total of 132 study participants; 82 participants with schizophrenia and 50 controls. hsCRP and IL-6 were measured using Cobas Integra 400 Plus and Cobas e 411 analysers respectively.

**Results:**

The levels of hsCRP and IL-6 were significantly increased among participants with schizophrenia compared to controls: hsCRP mean value 2.87 ± 5.6 vs 0.67 ± 0.6 mg/L; IL-6 mean value 6.63 ± 5.6 vs 3.37 ± 4.0 pg/ml. Controlling for potential confounders (age, sex and body mass index), having a diagnosis of schizophrenia remained significantly associated with increased hsCRP and IL-6.

**Conclusion:**

The results confirm that inflammatory processes may have a role in the pathophysiology of schizophrenia regardless of setting. Despite failure of some interventions with anti-inflammatory properties, interventions to reduce inflammation are still worth pursuing.

## Background

Schizophrenia is a relatively rare but serious mental disorder affecting about 1% of the adult population. Due to medical co-morbidities and other patient and service factors, people diagnosed with schizophrenia have a high overall mortality rate [1, 2], which may occur 20 to 30 years earlier than the general population [3]. The exact cause of schizophrenia is not established; however, epidemiological evidence indicates that several risk factors, including genetic susceptibility [4], season of birth [5], increasing parental age [6], and prenatal exposure to infection [7] may contribute to the development of schizophrenia.

Immunological dysfunctions, exposure to infectious agents that lead to immune response, such as *Toxoplasma gondii* [8], influenza [9] and interaction of environmental factors and stress are also considered risk factors [10]. Although casualty has not been proven, high C-reactive protein (CRP) and interleukin 6 (IL-6) concentration are reported in mental disorders [11].

CRP is nonspecific serum protein, traditionally considered as an acute phase immune response marker. It is mainly produced by liver cells and it is directly modulated by both interleukins (IL) 1β and IL-6, inflammatory markers increased during psychotic states [12–14]. There is high heterogeneity concerning the effect of antipsychotic medication on inflammatory markers. One study indicated that antipsychotic medications inhibit microglial activation, which is the source of pro-inflammatory markers, such as nitric oxide and TNF [15]. In a recent large longitudinal meta-analysis study that included 26 studies and 85,000 subjects, the serum levels of inflammatory markers were not affected with the introduction of antipsychotic medications [16].

A number of studies among patients with first episode and persistent or recurrent schizophrenia have shown increased serum levels of acute phase proteins, such as CRP, and proinflammatory markers such as tumor necrosis factor (TNF-alpha), IL-6, and IL-1β, although with some inconsistency [17–20]. A meta-analysis reported higher CR*P* values in patients with schizophrenia compared to that of the control group [13]. Two individual studies (case-control and longitudinal birth cohort study) indicated an association between increased CRP values and elevated risk of schizophrenia [21, 22]. On the other hand, few other studies have not found differences between serum CRP or IL-6 levels of patients with schizophrenia and control subjects [23, 24].

Most of these studies have been conducted in high-income countries, and although infectious causes may have more relevance in the causation of schizophrenia in low and middle-income countries. Therefore, this study aimed to investigate the serum level of inflammatory markers (CRP and IL-6) among patients with schizophrenia.

## Methods

### Participants

Eighty- two patients with schizophrenia according to the Diagnostic and Statistical Manual of Mental Disorders Fourth edition (DSM-IV) [25] were recruited between January 2015 and March 2016at the Amanuel Specialized Mental Hospital, the main national institution treating people with mental illness. Participants were recruited as part of a clinical trial, the MINOS (MINOcycline for Schizophrenia) Trial [26] (Clinicaltrials.gov identifier: NCT01809158). They were adults (≥ 18 years old), with a confirmed diagnosis of schizophrenia using the standardized evaluation (Operational Criteria for Research-OPCRIT) administered by a psychiatrist, and a recent onset of illness (duration under 5 years). The full detail of inclusion and exclusion criteria is described elsewhere [26]. To be included, participants were required to have a moderately severe illness (at least a score of 75 or more on the Positive and Negative Syndrome Scale (PANSS)), and should have been in receipt of antipsychotic treatment for at least 4 weeks with little response. Patients assessed by a physician to have any clinically significant or unstable medical disorder, including abnormal liver function or diseases, renal impairment, congestive heart failure, leukopenia, leucocytosis, anaemia, and thrombocytopenia were excluded. Additionally, patients recruited, did not have serious physical or neurological co-morbidity and did not abuse addictive substances. Considering the nature of the trial, women of child bearing age were excluded. For this study on inflammatory markers, the first 82 participants who provided blood sample were included. Fifty apparently healthy control subjects were recruited in the same geographic area as summarized in Table 1. Full medical and psychiatric assessments were conducted to the control subjects in order to exclude those with evidence of any acute or chronic general medical condition, and history of psychiatric illness that may affect the values of the inflammatory markers.

**Table 1:** Demographic and clinical Characteristics of study participants

Characteristics	Cases (Patients with Schizophrenia, ***n*** = 82)	Healthy Control Subjects (***n*** = 50)
Males/Females (n)	79 / 3	23 / 27
Age, Years Mean (SD)	35.1 (9.7)	28.8 (9.9)
BMI Mean (SD)	21.0 (3.35)	22.0 (2.8)
Ethnicity n (%)		
Oromo	11 (13.4)	n/a
Amhara	13 (15.9)	n/a
Tigray	8 (9.8)	n/a
Gurage	45 (54.9)	n/a
Others	5 (6.1)	n/a
Marital status n (%)		
Single	67 (81.7)	n/a
Married	6 (7.3)	n/a
Divorced	4 (4.9)	n/a
Widowed	5 (6.1)	n/a
Cohabiting	0	n/a
Living arrangement n (%)		
Lives alone	3 (3.7)	n/a
Lives with Parental family	63 (76.8)	n/a
Lives with Marital family	6 (7.3)	n/a
Lives with other relatives	10 (12.2)	n/a
Lives with friends	0	n/a
Age of onset in Years, Mean (SD)	22.88 (6.63)	n/a
PANSS total score Mean (SD)	89.2 (20.2)	n/a
PANSS classification in n (%)		
Markedly ill	50 (61.0)	n/a
Severely ill	22 (26.8)	n/a
Extreme severely ill	10 (12.2)	n/a
Duration of current episode		n/a
In months Mean (SD)	13.82 (18.2)	
Current Episode (%)		
First Episode	4.9	n/a
Relapse Episode	95.1	n/a
Smoking Status Yes/No	15/67	n/a

*BMI* Body mass index, *SD* Standard deviation,

*n/a* Not Available

### Assessments

A demographic questionnaire that included age, sex, and body mass index (BMI) was completed by a trained clinical nurse. Obesity classification was conducted using the BMI as follows: underweight (< 18.50), normal (18.5–24.99), overweight and obese (25 and above). The severity of the symptoms of schizophrenia was assessed using the PANSS, a widely used semi-structured instrument in schizophrenia research. The PANSS has been used successfully in a clinical trial in Ethiopia [27].

### Serum IL-6 and hsCRP measurements

Experienced phlebotomists collected 4–5 ml of blood from both study participants and control groups, after 8–10 h of overnight fasting. Samples centrifugation was performed at 5000 rpm on clotted blood for 10 min, and serum aliquots were stored at − 80°c until analysis, performed at the clinical chemistry laboratory of the Ethiopia Public Health Institute. hsCRP and IL-6 measurements were performed using turbidimetric and Electrochemiluminescence immunoassay methods with Cobas Integra 400 Plus and Cobas e411 (Roche Diagnostics GmbH, Mannheim, Germany) respectively. The lowest detectable limits for hsCRP and IL-6 were 0.1 mg/L and 1.5 pg/ml, respectively. IL-6 values of > 7 pg/ml and hsCRP values > 1 mg/L were considered high. The intra and inter-assay analytical coefficient of variations (CV_A_) for hsCRP using Precinorm Protein control was 1.2 and 1.3%, respectively. The intra and inter-assay CV_A_ for IL-6 using PreciControl multimarker 1 was 1.4 and 2.7%, respectively.

### Statistical analysis

Statistical analysis of the data was performed using SPSS Version 22.00 (SPSS Inc. Chicago, IL, USA). Simple descriptive and comparative analyses were carried out initially. For more advanced analysis, linear regression was used after evaluating the normality of the distribution of both hsCRP and IL-6. hsCRP was not normally distributed and thus was log-transformed. Gender, age, and BMI, factors previously reported to be associated with hsCRP and IL-6, were considered confounders and adjusted for in the linear regression model. All hsCRP and IL-6 values lower than the measuring range were coded as 0.1 and 1.5 respectively. All values of *p* < 0.05 were considered significant.

## Results

### Demographic and clinical characteristics

The socio-demographic characteristics of participants is presented in Table 1. Compared to the control group, participants with schizophrenia were predominantly male and slightly older. Over two-thirds of the patients with schizophrenia were single during the study period and lived with a parental family. Patients with Schizophrenia and control participants were similar in terms of current BMI. The mean PANSS score was 89.2 and over 61% were markedly ill as defined by their PANSS score.

### Immunological findings

**Fig. 1:**
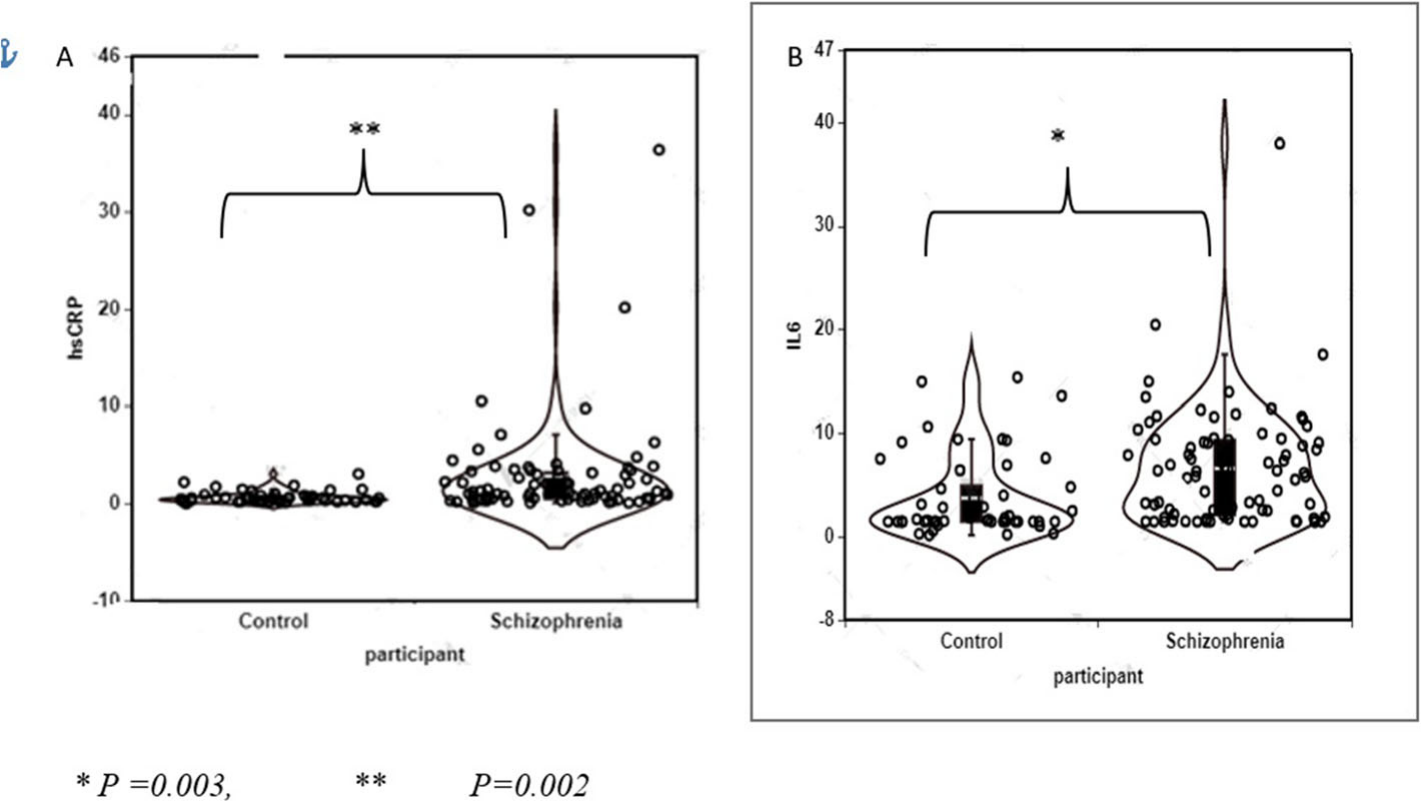
Scatter plots of serum hsCRP (**A**) and IL-6 (**B**) in schizophrenia and control group. *P* values derived after adjustment for gender, age, and BMI

Forty-nine percent and 43% of patients with schizophrenia had elevated hsCRP and IL-6 values, respectively. The mean value of hsCRP in patients with schizophrenia and controls was 2.87 ± 5.6 and 0.67 ± 0.6 mg/L respectively while the respective IL-6 values were 6.63 ± 5.6 vs 3.37 ± 4.0 pg/ml (Fig. 1). hsCRP level was statistically significantly associated with having a diagnosis of schizophrenia in both the crude (β =0.37, 95% CI = 0.20, 0.54, *p* < 0.001) and the adjusted (β =0.29, 95% CI: 0.10, 0.49, *p* = 0.003) models. Serum IL-6 was also statistically significantly higher among patients with schizophrenia in both the crude (β =2.86, 95% CI = 1.11, 4.62, *p* = 0.001) and the adjusted (β =3.60, 95% CI: 1.35, 5.86, *p* = 0.002) models (Tables 2 and 3).

**Table 2:** Comparison of the inflammatory markers (hsCRP, IL-6) between cases (participants with schizophrenia) and the control group

Marker Variables		Schizophrenia n (%)	Control group n (%)	χ^**2**^	df	***p***-value
hsCRP	Normal	42 (51.2)	42 (84.0)	14.423	1	< 0.001
	Elevated^a^	40 (48.8)	8 (16.0)			
IL-6	Normal	47 (57.3)	40 (80)	7.113	1	0.008
	Elevated^b^	35 (42.7)	10 (20)			

^a^hsCR *P* values of > 1 mg/L were considered elevated

^b^IL-6 values > 7 pg/ml were considered elevated

Other factors associated with hsCRP in the adjusted model were age, gender, and BMI (Table 3)

**Table 3:** Factors associated with serum level of hsCRP

Factors	Unadjusted hsCRP (log Transformed)				adjusted* hsCRP (log transformed)			
	β	95%	CI	P	β	95%	CI	P
Group								
Schizophrenia	0.37	0.20	0.54	**< 0.001**	0.29	0.10	0.49	**0.003**
Control	Ref.							
Gender								
Male	0.21	0.01 0.42		**0.041**	0.15	−1.08	0.38	0.206
Female	Ref.							
Age								
18–24 years	−0.45	−0.72	−0.17	**0.001**	−0.40	− 0.66	− 0.14	**0.003**
25–34 years	− 0.23	− 0.48	0.02	0.076	− 0.26	− 0.49	− 0.03	0.028
35–44 years	− 0.14	− 0.42	0.14	0.313	− 0.24	− 0.48	0.01	0.062
45+ years	Ref							
BMI								
Underweight	−0.53	−0.86	− 0.21	**0.001**	− 0.71	−1.02	− 0.41	**< 0.001**
Normal	− 0.40	− 0.67	− 0.13	**0.004**	− 0.40	−0.65	− 0.15	**0.002**
Overweight/Obese	Ref							

There were no associations between IL-6 and age of onset, duration of current episode, total PANSS score, and smoking status. Similarly, there were no associations between hsCRP and age of onset, duration of current episode, and smoking status. However, there was significant albeit negative association between PANSS score and hsCRP (β = − 0.011; 95% CI = − 0.018, − 0.004; *p* = 0.003) (Tables 4 and 5).

**Table 4:** Factors associated with serum level of IL-6

Factors	unadjusted IL-6				adjusted * IL-6			
	β	95%	CI	P	β	95%	CI	P
**Schizophrenia**	2.86	1.11	4.62	**0.001**	3.60	1.35	5.86	**0.002**
**Control**	Ref.							
**Gender**								
Male	1.16	−0.94	3.26	0.280	−0.92	−3.52	1.68	0.489
Female	Ref.							
**Age**								
18–24 years	−1.75	−4.67	1.17	0.241	−0.97	−3.95	2.01	0.524
25–34 years	− 1.00	−3.71	1.72	0.471	−0.76	−3.40	1.89	0.575
35–44 years	−1.46	−4.42	1.50	0.332	−1.76	−4.62	1.10	0.227
45+ years	Ref.							
**BMI**								
Underweight	−0.58	−4.04	2.89	0.744	−1.20	−4.69	2.30	0.503
Normal	0.27	−2.63	3.17	0.855	0.38	−2.50	3.26	0.797
Overweight/Obese	Ref.

**Table 5:** Associations between Clinical Characteristics of schizophrenia and inflammatory markers (hsCRP and IL-6)

Factors	IL-6				hsCRP log transformed			
	β	95%	CI	P	β	95%	CI	P
Age of onset of illness (Months)	0.02	−0.18	0.22	0.840	0.03	−0.02	0.02	0.783
Duration of current episode (Month)	0.02	−0.05	0.09	0.613	−0.0	−0.08	0.01	0.868
Duration of illness	−0.04	−0.20	0.13	0.673	0.014	0.00	0.028	0.058
PANSS	0.012	−0.01	0.09	0.776	−0.011	−0.018	− 0.004	0.003
Smoking	0.05	−0.23	0.33	0.730	0.06	−0.24	0.37	0.695

## Discussion

The main finding of the study is that significantly higher levels of both hsCRP and IL-6 were observed in patients with schizophrenia compared with the control group. To the best of our knowledge, this is the first study to investigate the serum level of both hsCRP and IL-6 among patients with schizophrenia in Ethiopia. It is also one of the very few studies from Africa. More broadly, diagnosis of inflammatory diseases and inflammatory markers in Africa is rare. Nevertheless, there is evidence of increase in the incidence and prevalence of some inflammatory diseases in the developing world, which may increase the significance of inflammation in neuropsychiatric syndromes.

The result of the present study concurs with studies from Western countries that consistently indicate that patients with schizophrenia have high serum levels of hsCRP and IL-6 [21, 28–32]. Elevated inflammatory markers in patients with schizophrenia have been reported in case control studies [21, 33] and treatment studies [16]. This is also found in people with both acute [29], chronic [30] and treatment-resistant [34] illnesses. Because of the consistency of this finding, neuro-inflammation has been linked with the causation of schizophrenia and other mental disorders. However, such studies are rare in low- and middle-income countries where the majority of the population of the world lives. We believe that this study contributes to this particular knowledge gap and the broader issue of lack of such studies even in the general population [35].

A significant negative correlation was observed between hsCRP and total PANSS score in our study. The evidence in the literature in this regard is mixed: some studies have reported negative correlation as observed in our study [36] while others have reported either positive association [33, 37] or no association between hsCRP and total PANSS score [38–41]. Despite these inconsistencies, hsCRP appears to be an important inflammatory marker in this particular setting although additional confirmatory studies would be needed.

The pathophysiology of schizophrenia has been linked with chronic inflammation, which stimulate inflammatory markers like CRP and IL-6 [42]. Both CRP and IL-6 have important roles in the inflammatory processes and CRP has been widely considered as a state marker along with other cytokines like TNF-alpha. CRP is an acute phase protein and produced by hepatocytes when stimulated by inflammatory markers including IL-6. Under normal conditions, CRP does not cross the blood-brain barrier. Increasing serum level of CRP may increase the permeability of blood-brain barrier by affecting the function of tight junction which facilities the entry of pro-inflammatory cytokines and CRP itself into the central nervous system. This would support the potential role of CRP in the pathophysiology of schizophrenia, Moreover, studies based on cell culture indicate that CRP can induce a pro-inflammatory state in microglia, thus suggesting that CRP may be linked to neuro- inflammation in the central nervous system [15, 43, 44].

## Conclusion

To the best of our knowledge, this is the first study that compares the inflammatory markers of patients with treatment-resistant schizophrenia with a control group. The result suggests that there is a higher level of hsCRP and IL-6 in patients with schizophrenia compared to their control groups. The cross-sectional design, the relatively small sample size, and the fact that study participants were not medication free are the potential limitations of this study. In conclusion, the result from this study along with others from developed countries, underscore the fact that inflammation plays an important role in the pathogenesis of schizophrenia globally.

## Data Availability

The datasets used and analyzed during the current study are available from the first author Feyissa Challa on reasonable request.

## Abbreviations

hsCRP: High sensitive C-reactive protein
IL-6: Interleukin-6
CRP: C-reactive Protein
TNF: Tumor necrosis factor
DSM-IV: Diagnostic and Statistical Manual of Mental Disorders Fourth edition
IRB: Institutional Review Board
BMI: Body mass index
